# Research progress on the roles of dopamine and dopamine receptors in digestive system diseases

**DOI:** 10.1111/jcmm.18154

**Published:** 2024-03-17

**Authors:** Xianmin Lu, Qi Liu, Ya Deng, Jiangbo Wu, Xingyi Mu, Xiaoxu Yang, Ting Zhang, Chen Luo, Zhuo Li, Siqi Tang, Yanxia Hu, Qian Du, Jingyu Xu, Rui Xie

**Affiliations:** ^1^ Department of Gastroenterology, Digestive Disease Hospital Affiliated Hospital of Zunyi Medical University Zunyi China; ^2^ The Collaborative InnovAffiliated Hospital of Zunyi Medical Universityation Center of Tissue Damage Repair and Regeneration Medicine of Zunyi Medical University Zunyi China

**Keywords:** brain‐gut axis, dopamine, dopamine receptor, liver cancer, neurotransmitter

## Abstract

Dopamine (DA) is a neurotransmitter synthesized in the human body that acts on multiple organs throughout the body, reaching them through the blood circulation. Neurotransmitters are special molecules that act as messengers by binding to receptors at chemical synapses between neurons. As ligands, they mainly bind to corresponding receptors on central or peripheral tissue cells. Signalling through chemical synapses is involved in regulating the activities of various body systems. Lack of DA or a decrease in DA levels in the brain can lead to serious diseases such as Parkinson's disease, schizophrenia, addiction and attention deficit disorder. It is widely recognized that DA is closely related to neurological diseases. As research on the roles of brain‐gut peptides in human physiology and pathology has deepened in recent years, the regulatory role of neurotransmitters in digestive system diseases has gradually attracted researchers' attention, and research on DA has expanded to the field of digestive system diseases. This review mainly elaborates on the research progress on the roles of DA and DRs related to digestive system diseases. Starting from the biochemical and pharmacological properties of DA and DRs, it discusses the therapeutic value of DA‐ and DR‐related drugs for digestive system diseases.

## 1 INTRODUCTION

**FIGURE 1:**
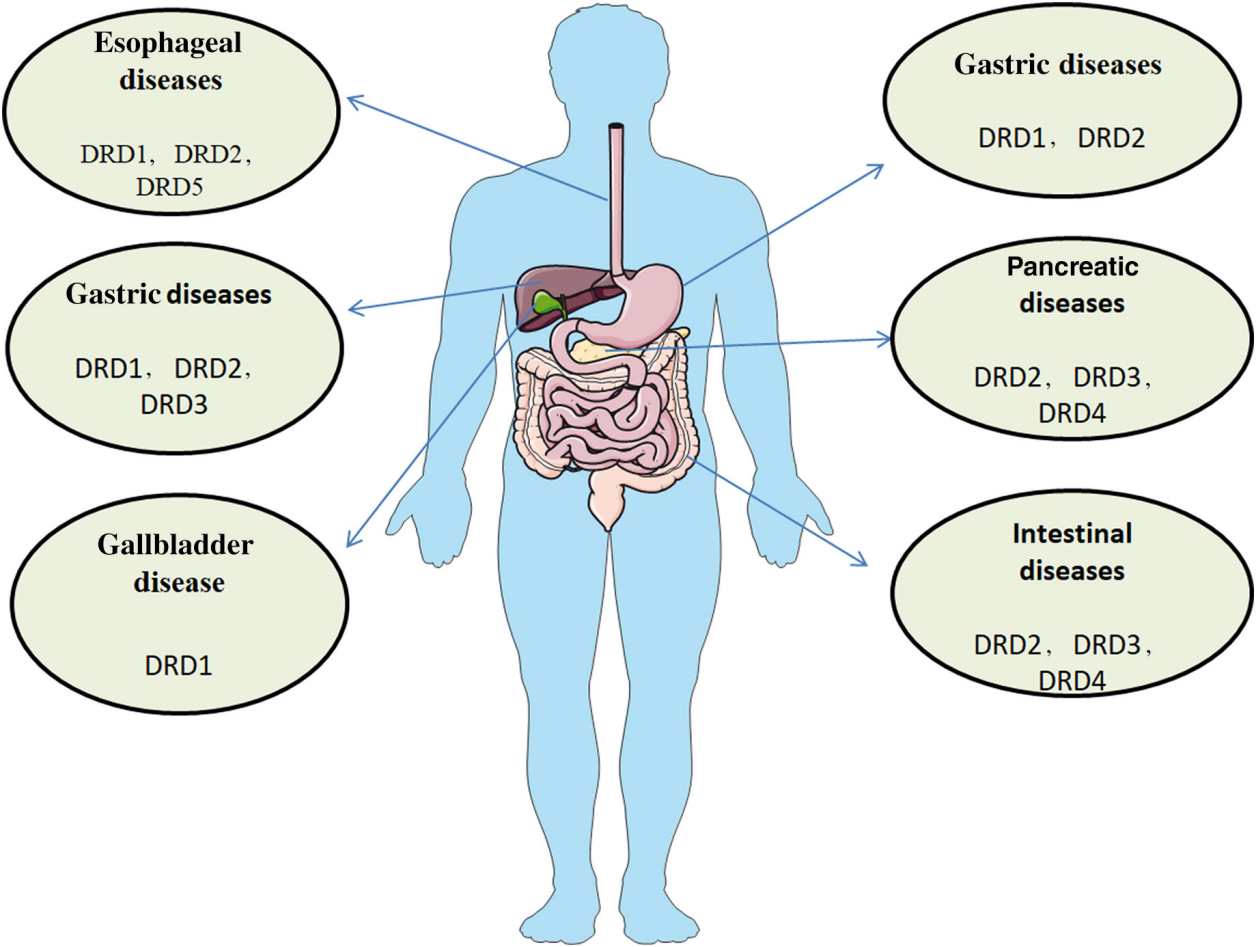
The tissue distribution of dopamine receptors in digestive system diseases.

DA is a neurotrans mitter synthesized in the central nervous system and peripheral nervous system. DA is a G protein‐coupled receptor ligand and exerts its effects by binding to specific membrane receptors.^1^, ^2^, ^3^, ^4^ DRs are classified into two categories based on their biochemical and pharmacological properties: D1‐like receptor subtypes Dopamine receptor D1 (DRD1) and Dopamine receptor D5 (DRD5), D2‐like receptor subtypes Dopamine receptor D2 (DRD2), Dopamine receptor D3 (DRD3), and Dopamine receptor D4 (DRD4).^5^, ^6^ In the periphery, different proportions of DR subtypes have been observed in the kidneys, adrenal glands, sympathetic glial cells, gastrointestinal tract (GIT), blood vessels and heart.^7^, ^8^ DA in plasma comes from dietary intake or endogenous synthesis. Endogenous DA is mainly produced by the brain, adrenal cortex, and intestines. The GIT is the main site of DA production in the body, and approximately 50% of DA is synthesized in the mesenteric organs of the human body.^9^, ^10^, ^11^ DA is involved in multiple physiological activities, such as food digestion, immune activation and regulation of intestinal endocrine signalling pathways in the intestine. In addition, DA receptors in the oesophagus, liver and pancreas are also involved in transmitting signals from the GIT and gut microbiota to the brain via the vagus nerve.^12^ Yan et al found that DA is an endogenous inhibitor of the activation of the NOD‐like receptor thermal protein domain associated protein 3 (NLRP3) inflammasome, a proinflammatory complex that plays an important regulatory role in immune function and participates in regulating the migration of white blood cells and tumour cells.^13^, ^14^ Research on the roles of DA and its receptors in the digestive system is increasing year by year. It was also found that dopamine (DA) receptors are expressed to a certain extent in various tissues and organs of the digestive system (Figure 1), Researchers are continuing to explore new aspects of the dopaminergic system and believe that DR modulators hold important clinical value for digestive system diseases (Table 1). However, at present, the understanding of the regulatory function of DR modulators in digestive system diseases and the related mechanism is still limited. This review summarizes the latest research on the roles of DA and DRs in digestive system diseases, hoping to provide research directions and references for the development of new DA modulators for the treatment of digestive system diseases.

**TABLE 1:** The application of dopamine receptor modulators in digestive system diseases.

Drug name	Regulatory action	Therapeutic effects	Reference
Domperidone	DRD2 antagonist	Promotes gastric peristalsis and emptying, in addition to inhibiting reflux of gastric contents after meals; can increase pressure on the lower oesophageal sphincter	[41, 90, 91]
Sulpiride	DRD2 inhibitor	Can reduce the secretion of gastric protein and gastric acid and inhibit the development of gastric ulcers	[18, 35, 42]
Cisapride	DRD2 antagonist	Can increase the release of acetylcholine in the intestinal plexus to stimulate the movement of the lower oesophagus, stomach, and small intestine	[19]
SKF 38393	DRD1 inhibitor	Inhibits the development of gastroduodenal ulcers	[35]
Metoclopramide	DR antagonist	Increases gastric emptying while also exerting antiemetic effects	[39, 40]
Domperidone	DRD2 antagonist	Inhibits intestinal motility, regulates sodium absorption and mucosal blood flow, and has a protective effect against gastroduodenal ulcers	[47, 48]
Calilazine	DRD3 antagonist	Can inhibit multiple drug resistance and make colon and lung cancer cells sensitive to antitumor drugs	[52]
Pimozite	DRD2 antagonist	Inhibits the growth and metastasis of colorectal cancer (CRC)	[53]
Thioridazine	DRD2 antagonist	Inhibits the growth of gastric cancer cells to exert antitumor effects; ameliorates hepatocellular carcinoma to possibly reduce the risk of tumour metastasis	[54, 83]
Quinpirol	DRD2 agonist	Reduces the permeability of blood vessels and prevents excessive leakage of blood vessels, thereby decreasing the severity of UC	[56, 57, 62]
Bromocriptine	**DRD2 antagonist**	Alleviates PUD and GERD	[62]
Cabergoline	A long‐acting DRD2 agonist	Ameliorates intestinal inflammation to significantly reduce ulcer formation, necrosis, and edema of the intestinal mucosa	[62]
Berberine	DRD1 and DRD2 antagonist	Alleviates the symptoms of colitis	[71]
Sunitinib	DRD2 inhibitor	Alleviates pancreatic cancer	[72]
Diphenylbutyl piperidine	DRD2 inhibitor	Alleviates pancreatic cancer	[73]
Rotigotine	Dopamine receptor agonist	Relieves pathological damage to liver tissue and reduces tumour necrosis factor‐α and interleukin‐6 levels	[81]
SCH23390	DRD1 inhibitor	Exerts antitumor effects both in vitro and in vivo	[84]

## 2 ROLES OF DOPAMINE AND DOPAMINE RECEPTORS IN OESOPHAGEAL DISEASES

As economic conditions have improved, the intake of a high‐fat and high‐sugar diet, smoking, alcohol consumption, etc., have become high‐risk factors leading to oesophageal diseases. The incidence rates of reflux esophagitis and oesophageal cancer, as oesophageal diseases, are increasing annually. Acid reflux, heartburn, hiccups and difficulty swallowing have become the main clinical symptoms of oesophageal diseases. Research has confirmed that vagal neurons, which transmit signals from the GIT and gut microbiota to the brain, innervate the oesophagus, with DA and DRs playing an important regulatory role at these synapses.^12^ A well‐developed ganglionated myenteric plexus (MP) is found in the oesophagus, and the highest number of neurons in the oesophagus is found in the MP.^15^ GERD is a common disease characterized by reflux of gastric contents into the oesophagus. Domperidone is a DRD2 blocker that promotes gastric peristalsis and emptying, inhibits reflux of postprandial gastric contents and can increase the tone of the lower oesophageal sphincter. Therefore, domperidone is a rational option for the treatment of gastro oesophageal reflux disease.^16^, ^17^ Sulpiride is a highly selective DRD2 antagonist mainly used for the treatment of PUD and GERD.^18^ Sisapride is a DRD2 antagonist. It can stimulate the movement of the oesophagus, stomach and small intestine.^19^ EC has become one of the most deadly cancers worldwide due to its invasiveness and low survival rate, and it has a poor prognosis.^20^ Qian et al. confirmed through in vitro and in vivo experiments that DA can stimulate the proliferation and growth of EC tumour cells through DRD5‐mediated pathways. Mechanistic research has revealed that activation of the DA pathway significantly increases glucose uptake and lactate production in oesophageal cancer EC tumour cells.^21^ DA and adenosine 3′5′‐monophosphate‐regulated phospho‐protein, Mr 32 kD (DARPP‐32) is a DA‐ and cyclic adenosine monophosphate (cAMP)‐regulated phosphoprotein found in DRD1 expressing dopaminergic neurons in the basal ganglia.^22^ Li et al. found through immunohistochemical analysis of DRD1 and DRD2 expression that DRD2/DARPP‐32 expression is associated with lymph node metastasis and tumour progression in oesophageal squamous cell carcinoma (ESCC) patients. DRD2/DARPP‐32 expression may help predict the prognosis of ESCC.^23^, ^24^ Additionally, Kline et al. found that DRD2 expression can be used to evaluate the prognosis of gastric or oesophageal cancer.^25^ Thus, DA and related receptors play an indispensable role in oesophageal diseases, and DR inhibitors have important therapeutic value in oesophageal‐related diseases.

## 3 THE ROLES OF DOPAMINE AND DOPAMINE RECEPTORS IN GASTRIC DISEASES

In recent years, the use of nonsteroidal anti‐inflammatory drugs, *Helicobacter pylori* infection, and poor dietary habits have led to an increase in the frequency of diseases such as gastritis, gastric ulcers and gastric cancer. DA and DRs play important physiological roles in the gastrointestinal system, with large amounts of DA found in the gastrointestinal mucosa of humans and mice.^26^, ^27^, ^28^ Feng et al. found that DA in the stomach is mainly secreted by parietal cells. After reaching the duodenal cavity, DA increases duodenal bicarbonate secretion (DBS) through DRD2‐ and calcium‐dependent pathways. Thus, it exerts a protective effect on the duodenal mucosa.^29^, ^30^ The large amount of endogenous DA produced in normal gastric tissue mainly regulates several physiological functions of the stomach, including reducing gastric acid secretion and stimulating bicarbonate and mucus secretion.^10^ DA is an important neurotransmitter and regulator in the epithelium.^31^ Normal, benign and malignant gastric tissues contain the high‐affinity DRD2. The use of DR antagonists to treat various stomach diseases is reasonable.^32^ Many studies have shown that the incidence rate of peptic ulcers in patients with DA deficiency is high, and DA over activity is related to a low incidence of ulcers. DA has a protective effect against the development of gastric ulcers.^29^, ^33^, ^34^ Desai et al. found through animal experiments that the DRD1 agonist SKF38393 has an inhibitory effect on gastric and duodenal ulcers in rats.^35^ Liu et al. found that DA and catecholamine metabolism are involved in gastric cancer through the DA transporter gene Solute Carrier Family 6 Member 3 (SLC6A3) and used real‐time fluorescence quantitative PCR (RT qPCR) to assess the expression of SLC6A3 in clinical samples and cells.

It has been confirmed that the expression level of SLC6A3 is significantly higher in gastric cancer patients than that in control subjects.^36^ Additionally, Mu et al. used Kaplan–Meier analysis to analyse the correlation between DRD2 expression and survival duration in gastric cancer patients. They found that the percentage of gastric cancer patients with high DRD2 expression levels (51.2%) was higher than that with low DRD2 expression levels (39.3%). Patients with higher expression of DRD2 had a shorter survival, and the expression of DRD2 was also negatively correlated with the prognosis of gastric cancer.^37^ Chakroborty et al. showed a decrease in DA levels in gastric cancer tissue and found that DA supplementation delayed the growth of gastric cancer by inhibiting angiogenesis.^38^ Domperidone and metoclopramide are commonly used drugs for the treatment of gastric diseases, and they alleviate gastric paresis, indigestion, chronic gastritis, gastric ulcers, and vomiting caused by various diseases. Domperidone and metoclopramide, as DR antagonists, can promote gastric emptying and exert antiemetic effects.^39^, ^40^ Domperidone also has a therapeutic effect on gastric palsy caused by Parkinson's disease.^41^ Additionally, oral administration of the DRD2 agonist sulpiride can reduce the secretion of gastric protein and gastric acid and inhibit the development of gastric ulcers.^35^, ^42^ In addition, the DRD2 antagonist thioridazine inhibits the growth of human gastric adenocarcinoma cell line (AGS) gastric cancer cells and exerts antitumor effects.^37^ In summary, DA and its receptors play an important regulatory role in gastric ulcers and gastric cancer, indicating that their mechanisms are complex and require further research.

## 4 ROLES OF DOPAMINE AND DOPAMINE RECEPTORS IN INTESTINAL DISEASES

Inflammatory intestinal diseases are often difficult to treat due to unclear aetiology. In recent years, considering the increased exploration of the relationship between the gut environment and the central nervous system, research on neurotransmitters and the gut has not been uncommon.^43^, ^44^ As mentioned above, besides the brain, the intestinal mucosa is the main peripheral organ that synthesizes DA. More than 50% of DA in the human body is synthesized in the intestine, and DA and DRs are widely distributed in the intestine, where they are mainly located in nerve endings.^10^, ^45^ DA in the intestine stimulates exocrine secretion and food digestion, inhibits intestinal motility and regulates sodium absorption and mucosal blood flow. DA plays an important regulatory role in these functions.^12^, ^46^, ^47^ The protective effect of DA can be significantly reversed by DRD2 antagonists, such as sulpiride and domperidone. DA in the GIT stimulates exocrine secretion, inhibits intestinal motility, regulates sodium absorption and mucosal blood flow, and has a protective effect against gastroduodenal ulcers. DA is a key neurotransmitter in the intestines that has been proven to regulate peripheral immune responses and to be associated with autoimmune diseases such as inflammatory bowel disease.^47^ A lack of DA in the intestine can lead to constipation.^48^ Endogenous DA mainly inhibits intestinal peristalsis through DRD2.^49^ By controlling intestinal peristalsis, DA can prevent the development of ulcers during intestinal injury. Magro et al. found that DA levels in the inflamed mucosa of Crohn's disease (CD) and ulcerative colitis (UC) patients were significantly reduced, while there was no change in DA levels in the noninflamed mucosa.^49^, ^50^ Through in vitro experiments, Al‐Jahmany et al. also confirmed that DA has an impact on ion transport in the distal colon of rats, especially the secretion of K^+^.^51^ Hussein et al. found that cariprazine is a DRD3 agonist that can inhibit multiple drug resistance and make colon cancer cells sensitive to antitumor drugs.^52^ Lee et al. found a positive correlation between increased expression of DRD2 and advanced colorectal cancer in patients, proving that DRD2 is activated in the disease. Chain protein Zinc Finger E‐Box Binding Homeobox 1 (ZEB1) signalling promotes the growth and migration of colorectal cancer (CRC) cells in vitro and in vivo. The DRD2 antagonist pimozide inhibits tumour growth and metastasis, indicating that DRD2 inhibitors are a potential therapeutic strategy for CRC.^53^ The DRD2 antagonist thioridazine (THIO) exerts antitumor effects in colorectal cancer.^54^ DA modulates the immune system by playing an important role in the differentiation of CD8^+^ cells into CD103^+^ TRM cells, thereby regulating antitumor immunity triggered by TRM cells in colorectal cancer.^55^ In inflammatory bowel disease, the DRD2 agonist quinpirol reduces vascular permeability and prevents excessive leakage, thereby decreasing the severity of UC.^56^, ^57^ Psychotherapy can help alleviate the symptoms of irritable bowel syndrome (IBS). Norepinephrine, 3‐hydroxytryptamine, and DA are crucial targets of drugs used to treat mental disorders and pain relievers.^58^ Miyazawa et al. found that DA may protect the small intestine from indomethacin‐induced damage by inhibiting DRD2‐mediated intestinal motility.^59^, ^60^ Berberine is a DRD1‐ and DRD2‐like antagonist. Kawano et al. found that berberine can alleviate colitis symptoms in a mouse model.^61^ Kurnik‐Lucka et al. constructed a mouse model of UC. Treatment with bromocriptine (a DRD2 subfamily agonist with high affinity for DRD2/3), quinpirol (a DRD3/4 agonist) and calergoline (a long‐acting DRD2 agonist) was found to significantly ameliorate intestinal inflammation and to significantly reduce ulceration, necrosis and edema of the intestinal mucosa in mice.^62^ Basu et al. found that the levels of DA, DRs and the second messenger cAMP were significantly reduced in human malignant colon tissues.^63^ According to these findings, DA serves as an important regulator in intestinal diseases, and DR inhibitors have important therapeutic value for intestinal diseases.

## 5 ROLES OF DOPAMINE AND DOPAMINE RECEPTORS IN BILIARY AND PANCREATIC DISEASES

DRD2 and DRD3 have been demonstrated by numerous studies to be expressed in the human pancreas. Both cells and adipocytes express DA, supporting its crucial role in regulating peripheral metabolism.^64^ When expressed in cells, these receptors are important negative regulators of insulin secretion and inhibit glucose‐stimulated insulin secretion.^65^, ^66^, ^67^ Insulin is a peptide secreted by the pancreas that plays an important role in regulating glucose metabolism in peripheral tissues. New evidence from human and animal studies suggests that insulin affects energy levels in the brain and increases synaptic activity, dendritic spine formation and the turnover of neurotransmitters such as DA.^68^ Yogo et al. constructed a mouse acute pancreatitis model and found that DA treatment inhibited the inflammatory response and reduced tissue damage in these mice.^69^ Somatostatin (SST) is the main drug used to treat pancreatitis. SST exerts its biological effects by interacting with specific receptors of the G protein‐coupled receptor superfamily, and DA is a G protein‐coupled receptor ligand. Lanrelin and octreotide are synthetic analogs of SST that have been widely used to treat pancreatic diseases.^8^ Through human studies, it has been found that DA secretion increases in patients with cholangiocarcinoma, and the increase in DA secretion by cholangiocarcinoma cells has a growth‐promoting effect on tumours. Inhibiting DA synthesis can reduce the proliferation of cholangiocarcinoma cells in vitro.^70^ In cholangiocarcinoma (BDC), DRD1 acts as a key protein involved in the proliferation of autonomous cancer stem cell‐like cells (CSCs) by regulating endogenous Wnt Family Member 7B (WNT7B). Drugs that inhibit DRD1 feedback signals combined with conventional chemotherapy may be a novel therapy for cholangiocarcinoma.^71^ Pancreatic cancer is a fatal malignant tumour with a five‐year survival rate. Studies have proven that sunitinib (SUN) is efficacious in treating pancreatic cancer. Moreover, DA significantly reduces the number of CSCs and increases the efficacy of SUN.^72^ Diphenylbutylpiperidine is an antipsychotic drug that is an effective inhibitor of DRD2 and is efficacious in treating pancreatic cancer.^73^ Researchers proved that in a mouse pancreatic cancer model, DA inhibits the M2 polarization of tumour‐associated macrophages (TAMs) and activates DRD4, leading to a decrease in cAMP levels, and then inhibits the activation of the protein kinase A (PKA/p38) signalling pathway, thereby inhibiting the tumour‐promoting inflammatory effects of TAMs. This reveals an interaction between TAMs and pancreatic cancer cells and suggests that DA exerts a synergistic effect with chemotherapy drugs for pancreatic cancer by inhibiting TAM‐mediated inflammation.^74^ DRD2 is a target for the treatment of pancreatic cancer. It was found that the expression level of DRD2 was increased in pancreatic cancer patients and that DRD2 inhibitors could slow tumour growth by inhibiting the extracellular regulated kinase (ERK) signalling pathway.^75^, ^76^ This indicates that DA is closely related to biliary and pancreatic diseases and that related drugs have important therapeutic value.

## 6 ROLES OF DOPAMINE AND DOPAMINE RECEPTORS IN LIVER DISEASE

Liver diseases, hepatitis, cirrhosis and liver cancer are all difficult to treat. In cirrhosis, it is difficult to reverse liver fibrosis. Most liver disease patients die from complications of liver cirrhosis. Hepatocellular carcinoma (HCC) is the sixth most common malignant cancer in the world and the third leading cause of cancer‐related deaths worldwide.^77^ Research on the connection between the liver and the neurotransmitter DA has recently been increasing year by year. Pacheco R, Contreras F et al. found that various cells in the liver, including dendritic cells, regulatory T cells, B cells and macrophages, and the autonomic nervous system can produce DA.^78^ Regulation of peripheral DA by the gut microbiota inhibits IL‐4 and interferon (IFN) secretion by invariant natural killer T (iNKT) cells γ and iNKT cell‐mediated hepatitis. The death of dopaminergic neurons significantly promotes the activation of liver iNKT cells and exacerbates liver injury induced by concanavalin A (ConA). DA plays an important role in suppressing autoimmune hepatitis.^79^ DRD2 antagonists normalize fibrotic macrophage‐endothelial cell crosstalk in nonalcoholic steatohepatitis, promoting liver regeneration rather than fibrosis in animal models.^80^ DR agonists have anti‐inflammatory effects. Rotigotine RoMS is a nonergot DR agonist of DRD2, DRD3 and DRD1. Pretreatment with a single dose of RoMS can inhibit LPS‐induced serum transaminase level elevation, alleviate liver tissue damage and reduce tumour necrosis factor‐α and interleukin‐6 levels.^81^ HCC remains one of the most common malignant tumours worldwide. Localized increases in DA secretion are seen in HCC. DA promotes the proliferation and metastasis of HCC cells. DRD1 is highly expressed in hepatocellular carcinoma tissue, and high expression of DRD1 is associated with poor prognosis in liver cancer patients.^82^ The DA level in the serum of HCC patients is lower than that in the serum of normal healthy individuals. However, treating HCC cells with DA can increase cell their proliferation, indicating that DA is an important factor in tumour growth. The DA antagonist thiazide may help reduce the risk of tumour metastasis to treat hepatocellular carcinoma.^83^ The local increase in DA secretion in HCC promotes the proliferation and metastasis of HCC cells. DRD1 has been found to protect against the progression of HCC and plays a crucial role in the DA system. SCH23390 is a selective DRD1 antagonist that exerts tumour‐inhibiting effects in vitro and in vivo. DRD1 may be a potential therapeutic target and prognostic biomarker for HCC.^84^ DA has recently been shown to be an inhibitor of the NLRP3 inflammasome and can treat ALF by inhibiting NLRP3 inflammasome activation and enhancing liver regeneration.^85^ Haak et al. constructed a fibrosis mouse model and found that DRD1 activation inactivated YAP/TAZ, switching activated fibroblasts from a fibrotic state of contraction, proliferation and matrix deposition to a matrix degradation‐promoting state, thereby reversing liver fibrosis.^86^ Zhao et al. found that DRD2 can be inhibited by TGF‐β1/Smarts and that the NF‐κB pathway partially reduces oxidative damage and the proliferation of hepatic stellate cells (HSCs) in diabetes.^87^ Yan et al. verified through in vivo experiments that DRD3 is associated with the prognosis of carcinoma in humans and inhibits the growth of tumour cells.^88^ Chen et al. validated the significant protective effect of DA against the pathogenesis of haematoxylin–eosin (HE) by constructing a fibrosis mouse model and using experimental techniques such as HE staining, protein immunoblotting, and immunohistochemistry.^89^ A decrease in DA expression is related to the pathogenesis of HE, and levodopa treatment significantly alleviates behavioural dysfunction in HE model rats. In addition, the researchers also found that the therapeutic effect of levodopa on behavioural impairment is mediated by DRD1.^89^ At present, there are no DR modulators used to treat diseases such as liver fibrosis, cirrhosis, liver cancer and hepatic encephalopathy in clinical practice, but research is ongoing.

## 7 CONCLUSIONS

In summary, the neurotransmitter DA and DRs play important roles in the pathogenesis, diagnosis and treatment of digestive system diseases. At present, the pathogenesis of digestive system tumours and inflammatory bowel disease is not clear, and the treatment of liver fibrosis, inflammatory bowel disease, autoimmune liver disease, pancreatic cancer, liver cancer and other diseases is still difficult. The lack of specific therapeutic and preventive drugs presents a challenge in clinical practice. With the emergence of research on the ‘brain‐gut axis’, it has been found that the neurotransmitter DA is not only closely related to the physiological functions of the nervous and cardiovascular systems but also closely related to digestive system diseases. Recently, promoting ‘old drugs for new use’ has become a trend in research, greatly reducing research costs. This article mainly summarizes the role of DA and DRs in the digestive system, focusing on the application of DA and DR inhibitors and agonists as therapeutic drugs for digestive system diseases. Nevertheless, developing new DA and DR modulators remains a challenging task. Although scientists have achieved successful breakthroughs in drug development, most DA inhibitors have only been used successfully in animal models and have not yet been used clinically. The application of newly developed DA modulators in clinical practice remains an enormous challenge. At present, researchers are limited to studies on the effectiveness of DA inhibitors in the treatment of related gastrointestinal diseases, and research on how the mechanisms underlying the effects of the drugs is lacking. To enable newly developed DA and DR modulators to be applied in clinical practice, close collaboration among multidisciplinary scientists is needed, as well as the participation of clinicians to gain a deeper understanding of the functions of DA and DRs and the related mechanisms and to further reveal the connection among DA, DRs and digestive system diseases. This review acts as a reference for researchers, identifying DA and DA modulators as therapeutic targets for digestive system diseases, providing new ideas and research directions for the diagnosis, prevention and treatment of these diseases.

## AUTHOR CONTRIBUTIONS

**Xianmin Lu:** Writing – original draft (equal). **Qi Liu:** Writing – original draft (equal). **Ya Deng:** Resources (equal). **Jiangbo Wu:** Resources (equal). **Xingyi Mu:** Resources (equal). **XiaoXu Yang:** Resources (equal). **Ting Zhang:** Resources (equal). **Chen luo:** Resources (equal). **Zhuo Li:** Resources (equal). **Siqi Tang:** Resources (equal). **YanXia Hu:** Resources (equal). **Qian Du:** Resources (equal). **JingYu Xu:** Supervision (equal); writing – review and editing (equal). **Rui Xie:** Supervision (equal); writing – review and editing (equal).

## FUNDING INFORMATION

This study was supported by research grants the National Natural Science Foundation of China (no. 81660099; no. 82170628; no. 81970541; no. 31960151; no. 32160208; no. 81770610) and Collaborative Innovation Center of Chinese Ministry of Education (2020‐39). This study was supported by the Outstanding Scientific Youth Fund of Guizhou Province (Qian Ke He Platform Talents [2021] 5647). This study was supported by Key Projects of Guizhou Provincial Basic Research Program (QIAN KE HE JI CHU‐ZK (2021) ZHONG DIAN 004). This study was supported by Zunyi Science and Technology Bureau (Outstanding Young Talents in Zunyi City (2020‐1)); This study was supported by Graduate Education and Teaching Innovation Program of Zunyi Medical University (ZYK163) and the Science and Technology Plan Project of Guizhou Province (QIANKEHEJICHU‐ZK(2023)YIBAN556).

## CONFLICT OF INTEREST STATEMENT

The authors declare that they have no competing interests.

## CONSENT FOR PUBLICATION

We have obtained consents to publish this paper from all the participants of this study.

## Data Availability

Not applicable, all information in this review can be found in the reference list.
